# Regression models from portable NIR spectra for predicting the carcass traits and meat quality of beef cattle

**DOI:** 10.1371/journal.pone.0303946

**Published:** 2024-05-31

**Authors:** Nathália Veloso Trópia, Rizielly Saraiva Reis Vilela, Flávia Adriane de Sales Silva, Dhones Rodrigues Andrade, Adailton Camêlo Costa, Fernando Alerrandro Andrade Cidrini, Jardeson de Souza Pinheiro, Pauliane Pucetti, Mario Luiz Chizzotti, Sebastião de Campos Valadares Filho

**Affiliations:** Department of Animal Science, Universidade Federal de Viçosa, Viçosa, Minas Gerais, Brazil; University of Illinois, UNITED STATES

## Abstract

The aims of this study were to predict carcass and meat traits, as well as the chemical composition of the 9th to 11th rib sections of beef cattle from portable NIR spectra. The 9th to 11th rib section was obtained from 60 Nellore bulls and cull cows. NIR spectra were acquired at: P1 –center of *Longissimus* muscle; and P2 –subcutaneous fat cap. The models accurately estimated (P ≥ 0.083) all carcass and meat quality traits, except those for predicting red (a*) and yellow (b*) intensity from P1, and 12th-rib fat from P2. However, precision was highly variable among the models; those for the prediction of carcass pHu, 12^th^ rib fat, toughness from P1, and those for 12^th^ rib fat, a* and b* from P2 presented high precision (R2 ≥ 0.65 or CCC ≥ 0.63), whereas all other models evaluated presented moderate to low precision (R2 ≤ 0.39). Models built from P1 and P2 accurately estimated (P ≥ 0.066) the chemical composition of the meat plus fat, bones and, meat plus fat plus bones, except those for predicting the ether extract (EE) and crude protein (CP) of bones and the EE of Meat plus bones fraction from P2. However, precision was highly variable among the models (–0.08 ≤ R2 ≤ 0.86) of the 9th and 11th rib section. Those models for the prediction of dry matter (DM) and EE of the bones from P1; of EE from P1; and of EE, mineral matter (MM), CP from P2 of meat plus fat plus bones presented high precision (R2 ≥ 0.76 or CCC ≥ 0.62), whereas all other models evaluated presented moderate to low precision (R2 ≤ 0.45). Thus, models built from portable NIR spectra acquired at different points of the 9th to 11th rib section were recommended for predicting carcass and muscle quality traits as well as for predicting the chemical composition of this section of beef cattle. However, it is noteworthy, that the small sample size was one of the limitations of this study.

## 1 Introduction

Estimating nutrient requirements faces a significant challenge in quantifying body composition. The direct determination of body composition is the most accurate method available, generating highly reliable data. However, its adoption as an experimental routine is impractical, as this process is lengthy, laborious, and expensive because of the laboratory analyses involved and the fact that at least half of the carcass cannot be marketed. Thus, faster and more economical methods for estimating body composition are required. In this context, Hankins and Howe [1] proposed an indirect method to estimate the carcass composition of cattle based on the composition of the section between the 9th and 11th ribs. This indirect method has been modified over the years to better estimate the carcass composition as well as the whole-body composition of cattle of different genotype and sexual classes [2–4]. Nevertheless, this indirect approach requires the complete dissection and analysis of the chemical composition of the 9th to 11th rib section tissues, as well the determination of other predictive variables such as the proportion of organs and viscera and/or visceral fat in the animal body, which can complicate the prediction of body composition.

A decisive factor in improving the beef market is assuring the quality of the meat, not only because this directly affects the profit of slaughterhouses and producers, but also because it is closely related to the consumer’s perception of the beef. Some variables can be evaluated to determine carcass traits and meat quality, such as, carcass yield, subcutaneous fat thickness, pHu, water loss during cooling, color, and toughness [5–7]. Nonetheless, like the determination of the chemical composition of the carcass, the evaluation of carcass traits and meat quality still depends on sampling and conventional laboratory analyses.

Near-infrared spectroscopy (NIR), along with chemometric techniques, is an alternative to conventional analyses, which among other major drawbacks, are usually destructive and polluting due to the use of different chemical reagents. Furthermore, predicting the chemical composition, carcass traits and meat quality by NIR spectra acquisition can reduce meat waste in the slaughterhouse, as well as reduce the time required to obtain the results. In fact, there has been growing interest in developing different models to predict the composition of carcass tissues [8–10], as well as the carcass and meat quality attributes [8, 9, 11–13] of different animal species. However, these studies acquired spectra from meat samples that were processed or removed from the carcass [14–16], making this process both lengthy and expensive.

In this context, the development and evaluation of predictive equations from NIR spectra acquired in easily accessible *in natura* tissues, with no processing required, such as in the intact backfat and longissimus muscle, should provide a useful tool for predicting the chemical composition of the 9th to 11th rib section, as well as the carcass traits and meat quality of beef cattle. Thus, we hypothesized that predictive models from portable NIR spectra acquired from the intact backfat, and longissimus muscle could replace conventional analyses of the chemical composition of the section between the 9th and 11th ribs, as well those for determining the carcass traits and meat quality of beef cattle. The objectives of this study were 1) to develop and evaluate the goodness of fit of regression models for predicting carcass traits and meat quality of beef cattle from portable NIR spectra acquired from the intact backfat and longissimus muscle and 2) to develop and evaluate the goodness of fit of regression models from portable NIR spectra acquired from the intact backfat and longissimus muscle to estimate the chemical composition of the section between the 9th and 11th ribs of beef cattle.

## 2. Material and methods

### 2.1. Animals, slaughter procedures, and sample and data collections

Sixty 9th to 11th rib sections were collected from 60 Nellore cattle of different categories (36 bulls and 24 cull cows), with an average shrunk body weight (SBW) of 389 ± 12.5 kg for bulls and 508 ± 11.5 kg for cull cows, and an average age of 15±0.17 months for bulls and 72±0.68 months for cull cows, slaughtered at the Frigorifico Escola of the Department of Animal Science at the Universidade Federal de Viçosa (UFV), Viçosa, Minas Gerais, Brazil (Table 1). All procedures involving care and handling of animals were approved by the Ethics Committee in the Use of Animals of the Universidade Federal de Viçosa (protocol n° 044/2021, and, 032/2021).

**Table 1:** Descriptive of the overall data base used.

Number of animals	Category	Average age (months)	Standard deviation of age	Average Weight (kg)	Standard deviation of Weight
36	Bulls	15	0.17	389	12.46
24	Cull Cows	72	0.68	508	11.47

After slaughter, the carcass of each animal was divided into two halves that were weighed (hot carcass weight; HCW) and cooled at –4°C for 24 h. After this time, the carcass was weighed again (cold carcass weight; CCW) and the pHu and 12th-rib fat were measured between the 12th and 13th ribs on the left half-carcass. The pHu was recorded using an electrode (model: FC2023 HANNA^®^ instruments, Barueri, Brazil) connected to a portable HANNA^®^ instruments pHu meter. Then, the cooling losses (CL) were calculated, which represented water loss (%) by dripping and evaporation of the carcass in the cold chamber = CL (%) = [(HCW—CCW)/ HCW] × 100.

**Fig 1:**
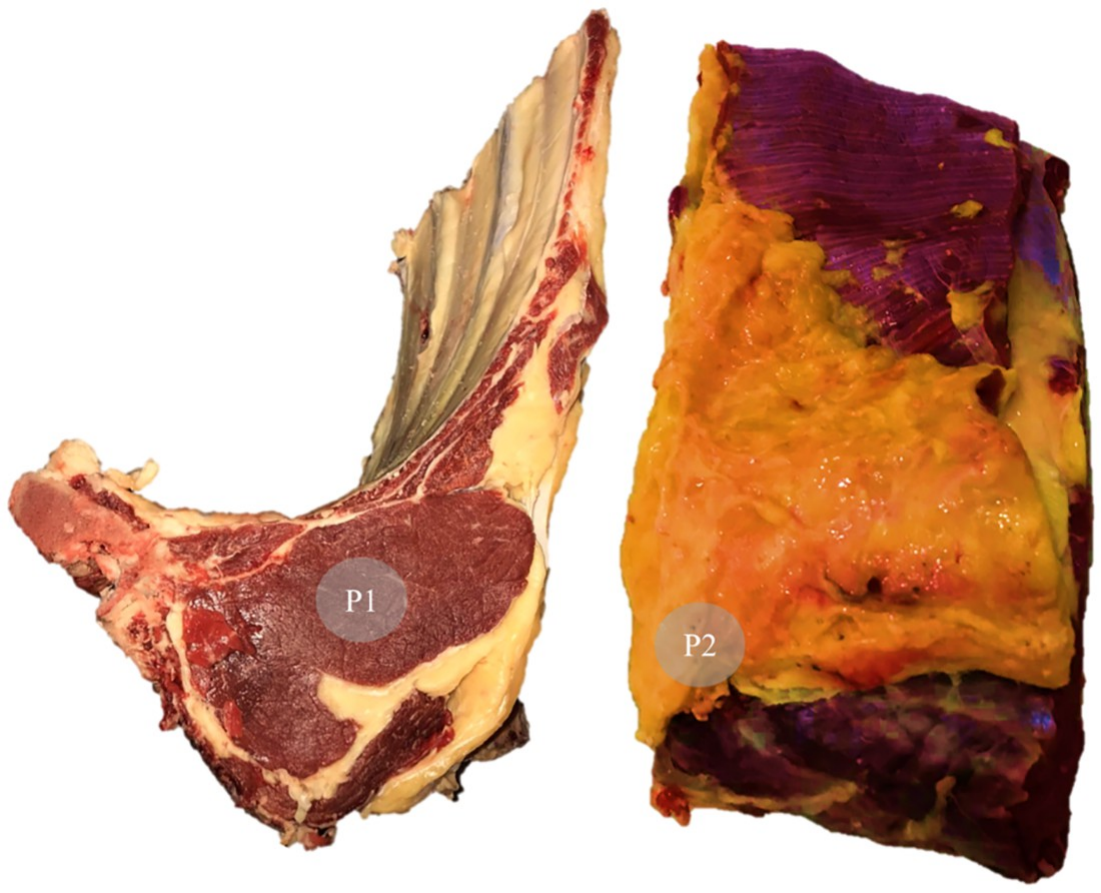
Points of spectra acquisition in the section between the 9th and 11th ribs of beef cattle *in natura*. Representation of the points where NIR spectra were acquired in the section between the 9th and 11th ribs of beef cattle *in natura*. P1 = spectra acquired on the center of the transversal cut of *Longissimus dorsi* muscle; and P2 = spectra acquired on the subcutaneous fat cap.

The 9th to 11th rib section was obtained from the left half-carcass, as recommended by [1], for subsequent acquisition of NIR spectra. The spectra were acquired using a portable NIR device (ITPhotonics S.r.l., model poliSPECNIR 900–1700, Breganze, Italy) with the aid of the poliDATA software (ITPhotonics S.r.l, Breganze, Italy) at two points of the section between the 9th and 11th ribs *in natura* (Fig 1): P1 –spectra acquired from the center of a transverse section of the *Longissimus dorsi* muscle; and P2 –spectra acquired from the subcutaneous fat cap. Five spectral readings were taken per acquisition point (P1 or P2). For further analysis, the average of the five spectra per acquisition point for each sample was calculated. Fig 1 illustrates the average spectra utilized in constructing the regression models. A small amount of pressure was applied during the spectra acquisition to ensure the compactness of the matrix analyzed in the scanning window. The spectra were collected in an air-conditioned room (average temperature of 10°C). Thus, the spectra of each point evaluated in each 9^th^ to 11^th^ rib section were obtained and recorded as log (1/R), where R is the reflectance of the sample, in the range of 884.9 and 1702.9 nm, measured at intervals of 3.2. The portable NIR device presented a measurement area of approximately 290 mm^2^ and its calibration was performed with default settings.

Then, *Longissimus dorsi* muscles were sampled between the 12th and 13th ribs from the left half-carcass. *Longissimus dorsi* samples were vacuum-packed, frozen and stored at −20°C until further meat quality analyses. Two 2.54 cm thick steaks were obtained from each *Longissimus dorsi* muscle sample, one for the instrumental color measurement, the second for estimations of thawing and cooking losses (total losses) followed by Warner-Bratzler shear force determinations. All analyses were performed at the Meat Science Laboratory (Laboratório de Ciência da Carne—LCC) of the Department of Animal Science at the Universidade Federal de Viçosa, Viçosa, Minas Gerais, Brazil.

Steaks were thawed for 16 hours at 4°C, removed from the vacuum packaging and exposed to oxygen at room temperature for 30 minutes prior to measurements. Instrumental color readings were obtained using a Hunter MiniScan EZ (4500L; Hunter Associates Laboratory, Inc., Reston, Virginia, USA), which was calibrated immediately prior to data collection. The mean L* (lightness), a* (intensity of red), and b* (intensity of yellow) values of each steak were determined from five readings on the steak surface using illuminant D65, a 31.8 mm port size and a 10° standard observer [17].

The analysis of total losses (thawing and cooking losses) and shear force was performed following the protocol proposed by Bruce et al. [18]. Briefly, steaks were thawed at 4°C for 16 h and cooked in a preheated water bath at 70°C for 40 min (model NT 268, Novatecnica, Piracicaba, SP, Brazil) The steaks were weighed before and after thawing and cooking to obtain the losses, which were expressed as a percentage of the steak weight prior to these procedures.

Cooked steaks were cooled for 16 h at 4°C prior to the removal of five cores (1.27 cm diameter) from each steak parallel to the longitudinal orientation of the muscle fibers. Each core was sheared once, perpendicular to the longitudinal orientation of the muscle fibers using a V-cut blade, with an angle of 60° and a thickness of 1.016 mm, and a fixed speed of 2mm/s, coupled to a Warner-Bratzler texturometer (G-R Eletrical Manufacturing Company, Manhattan—KS, USA). The shear force was expressed in Newton (N).

The sections between the 9th and 11th ribs collected from Nellore cull cows and bulls were obtained from the left half- carcass, as recommended by [1], and subsequently dissected into bone and meat plus fat tissues. Dissected samples were weighed, individually ground, and samples of approximately 300 g on a fresh weight basis were taken, packed in aluminum trays, and lyophilized.

All lyophilized samples were ground in a knife mill (Fortinox/model STAR FT-80, Piracicaba, Brazil) using liquid N. Thus, two sub-samples per heifer, named “meat plus fat-rib section” and “bones-rib section” were taken for further determination of the chemical composition. The meat plus fat-rib section was comprised of muscle and fat from the 9th to 11th rib section, and the bones-rib section contained the bone sample from the 9th to 11th rib section.

Meat plus fat-rib section and bones-rib section samples were analyzed for dry matter (DM; [19]; method 934.01), crude protein (CP; method 954.01), mineral matter (MM; method 930.05), and EE (method 920.39) content [20]. The CP, MM and EE contents of the whole section between the 9th and 11th ribs (meat plus fat plus bones) were mathematically estimated from the physical proportions of each fraction (meat plus fat and bones) and their respective chemical compositions.

### 2.2. Statistical and chemometric analyses

**Fig 2:**
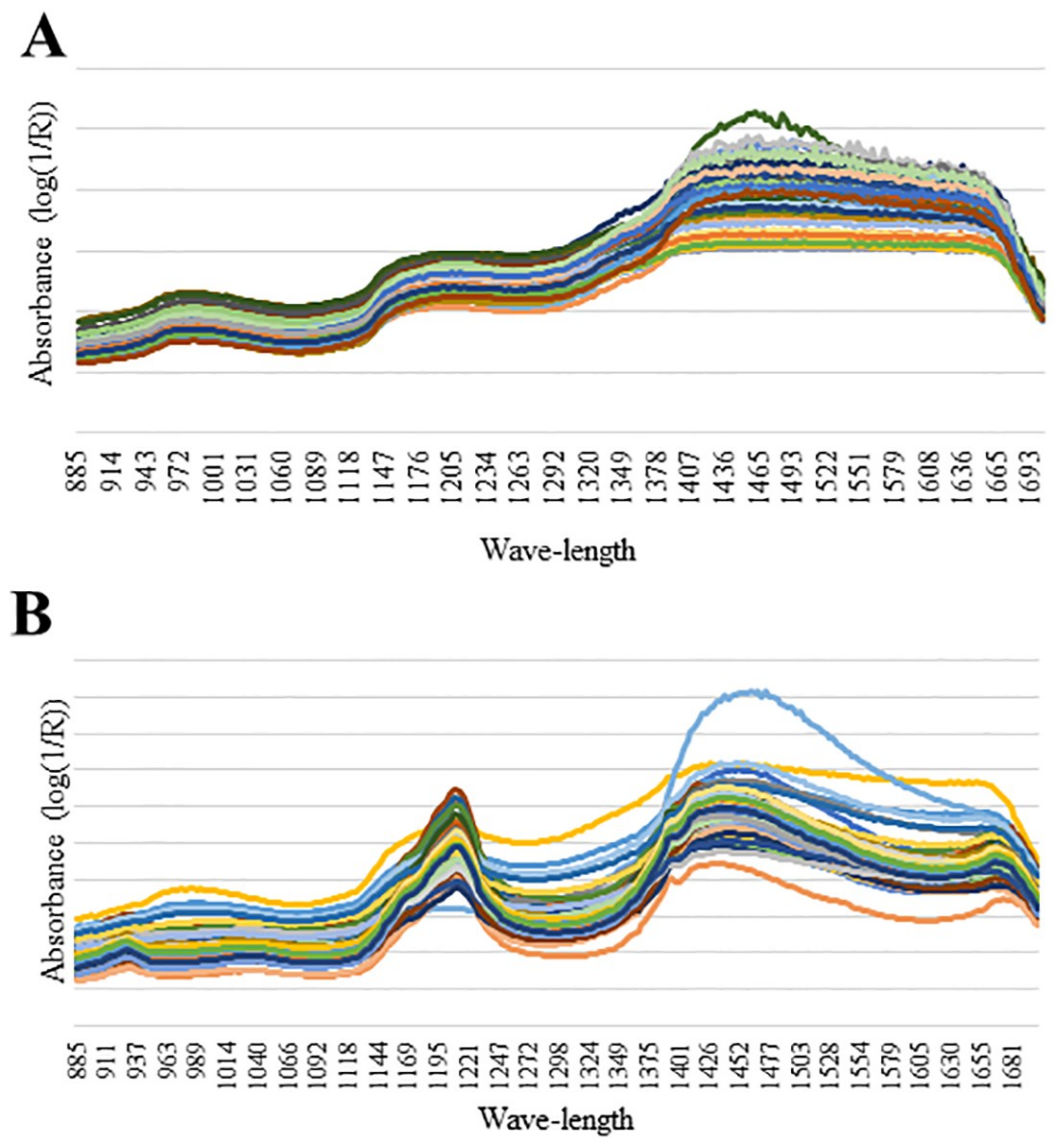
Spectral data acquired of the section between the 9th and 11th ribs of beef cattle *in natura*. Spectral data acquired at P1 (A) and P2 (B) of the section between the 9th and 11th ribs of beef cattle in natura. P1 = spectra acquired on the center of the transversal cut of Longissimus dorsi muscle; and P2 = spectra acquired on the subcutaneous fat cap.

The X matrices were constructed for each data set (1 –carcass traits and meat quality and 2—chemical composition of the section between the 9th and 11th ribs) and spectra acquisition point (P1 and P2), which contained the information for the independent variables. Each row of the X matrices corresponded to a sample and each column corresponded to an absorbance value for a given wavelength. The Y vectors were also created for each data set, as follows: 4-four vectors associated with the chemical composition of the meat plus fat and bones fractions (DM, MM, CP and EE) of the 9th to 11th rib section; three vectors associated with the chemical composition of the, meat plus fat plus bones (CP, MM and EE) of the 9th to 11th rib section; and eight vectors associated with the carcass traits and meat quality (pHu, 12th-rib fat, L*, a*, b*, CL, total loss and toughness). The spectra used to develop the models from P1 and P2 can be seen in Fig 2.

The files containing the spectra of each dataset that were assigned for the development of models were imported into the PLS-toolbox 8.2.1 software, which operates in a Matlab 2019b environment (Math Works, Natick, USA). Initially, outlier removal was performed using partial least squares (PLS) applied to the spectra. The sets of corresponding X and Y matrices were removed when they were detected as outliers based on graphical analysis of Hotelling’s T^2^ vs Reduced Residual Q and Leverage vs. Student Residual Y tests, following the methods described by Peternelli et al. [21] and Montgomery [22]. Subsequently, the data set collected for predicting carcass and meat quality traits was divided into two sub datasets, 75% of the data being used for model development and 25% of the data for external evaluation using the Kennard-Stone algorithm [23], which selects samples based on their distances.

The multivariate calibration method through partial least squares (PLS) regression was used for the development of all models. Leave one-out cross validation was used. Besides the raw data, two preprocessing methods (mean center and autoscaling) and eight transformations (smoothing, first derivative, second derivative, multiplicative scattering correction/signal correction [DMC], detrend, normalize, baseline correction, standardized signal normalization [SNV]; [24]) and their combinations (pre-treatments) were tested to find the best regression model.

The quality of the models was evaluated using the root mean square error of cross-validation (RMSECV) and correlation coefficient (RCV). The best model for each variable evaluated was chosen based on the lowest RMSECV. Furthermore, the best model (based on the lowest RMSECV) for predicting each carcass and meat quality variable, was submitted to external evaluation. For this, the carcass and meat quality variables estimated by portable NIR equations and observed values were evaluated according to the following regression model: y = β0 + β1 × x, where x = predicted values; y = observed values; β0 and β1 = intercept and slope, respectively. The regression was evaluated according to the following statistical hypothesis: H0: β0 = 0 and H0: β1 = 1, and Ha: not H0.

The goodness-of-fit of the calibration models was further evaluated using: the coefficient of determination (R2) which is the linear correlation between the predicted and observed values; the correlation and concordance coefficient or reproducibility index (CCC) described by Tedeschi [25]; and the mean square error of prediction (MSEP) and its components [26]: squared bias (SB), component relative to the model of random fluctuation (MoF), and magnitude of random fluctuation (MaF). For comparisons, *p-value* of 0.05 was established as the critical level of probability for type I error. All calculations were obtained using the Model Evaluation System (MES; [25]).

## 3. Results

### 3.1. Carcass and meat quality attributes

For all carcass and meat quality attributes, the regression models obtained utilizing pre-treated spectra presented the lowest values of RMSECV, indicating the best model fit and precision (Table 2). Autoscaling pre-treatment was used in five of the eight models built to predict carcass and meat quality attributes from the spectra acquired at P1; whereas normalize combined with second derivative were used in three of the eight models built from the spectra obtained at P2.

**Table 2:** Information on data sets used to develop and the respective performance parameters of PLS models to predict carcass traits and meat quality of beef cattle from spectra acquired at P1 and P2^1^.

Spectra acquisition^1^	Parameters^2^	Pre-treatments^3^	Matrix size	nVL^4^	RMSECV^5^	RCV^6^
P1	pHu	Au, + Su,	56x256	4	0.15	0.61
	12 th -rib fat	Norm, + DMC	52x256	4	1.91	0.74
	L*	Au, + Su,	40x256	6	4.08	0.70
	a*	SNV + Au,	40x256	4	3.87	0.74
	b*	Au, + Det,	40x256	3	4.98	0.45
	CL	1^a^d + LB	57x256	2	0.31	-0.05
	Total losses	Det, + CM	55x256	3	4.46	0.36
	Toughness	Au, + SNV	55x256	1	1.39	0.10
P2	pHu	Norm, + 2^a^d	58x256	1	0.20	-0.30
	12 th -rib fat	Su, + 2^a^d	57x256	7	2.30	0.63
	L*	Su,	42x256	2	5.54	0.02
	a*	Norm, + Det,	42x256	5	4.26	-0.11
	b*	DMC + Det,	41x256	3	3.44	-0.13
	CL	Norm, + Det,	57x256	2	0.20	0.16
	Total losses	2^a^d + LB	54x256	4	3.83	0.27
	Toughness	Su, + DMC	52x256	2	1.06	0.37

^1^P1 = spectra acquired on the center of the transversal cut of Longissimus dorsi muscle; and P2 = spectra acquired on the subcutaneous fat cap;

^2^pHu = carcass pHu 24 h after slaughter, L* = lightness, a* = red intensity, b* = yellow intensity, CL = cooling losses;

^3^Au. = autoscaling, 1st d = first derivative, Su. = smoothing, Norm. = normalization, DMC = multiplicative scattering correction, 2nd d = second derivative, LB = baseline correction, SNV = standardized signal normalization, Det. = detrend, CM = center on the mean;

^4^number of latent variables;

^5^root mean square error of the cross-validation;

^6^cross-validation correlation coefficient.

The models developed for estimating the 12th-rib fat, a*, b*, and toughness from spectra acquired at both P1 and P2, as well as those models predicting CL and total losses from spectra acquired at P1 presented moderate to high values of RMSECV in relation to the respective mean (20% ≤ RMSECV ≤ 51%). Others carcass and meat quality attributes evaluated (pHu and L* from spectra acquired at P1 and P2; and CL and total losses from spectra acquired at P2) showed low values of RMSECV (2% ≤ RMSECV ≤ 19%).

The predictive models for pHu, 12th-rib fat, L*, and a* from P1 spectra and 12th-rib fat from P2 spectra showed moderate to high precision in the cross-validation (0.61 ≤ RCV ≤ 0.74). Moderate precision (0.42 ≤ RCV ≤ 0.53) in the cross-validation was observed when b* was estimated from spectra acquired at P1. However, low precision (RCV ≤ 0.37) was observed for the predictive models for CL, total losses and toughness from P1 spectra, as well as for pHu, L*, a*, b*, CL, total losses, and toughness from P2 spectra.

External evaluation analysis showed that predictive models developed from spectra acquired at P1 accurately estimated all carcass and meat quality attributes, except the a* and b* attributes, as the null hypothesis of a respective intercept and slope equal to 0 and 1 (P > 0.05) was not rejected (Tables 3 and 4). Furthermore, the models built from the spectra acquired at P2 accurately estimated all carcass and meat quality attributes, except 12th-rib fat, as they did not reject (P ≥ 0.144) the null hypothesis of a respective intercept and slope equal to 0 and 1.

**Table 3:** Mean and descriptive statistics for the relationship between the observed and predicted values of carcass traits and meat quality of beef cattle.

Parameters^1^	SA^2^	Items^3^	Average (%)	SD^4^	Maximum	Minimum	R2^5^	CCC^6^	Regression: Intercept			Regression: Slope			MSEP^10^	QV^11^	MaF^12^	MoF^13^
									Estimated	SE^7^	P-value^8^	Estimated	SE^9^	P-value				
pHu	P1	Obs	5.8	0.21	6.2	5.5	-	-	-	-	-	-	-	-	-	-	-	-
		Au + Su	5.7	0.24	6.3	5.4	0.65	0.81	1.58	0.84	0.083	0.73	0.15	0.088	0.018	0.001	0.004	0.013
	P2	Obs	5.7	0.17	6.2	5.5	-	-	-	-	-	-	-	-	-	-	-	-
		Norm + 2^a^d	5.7	≤0.01	5.7	5.7	-0.06	0.01	-19.21	60.19	0.755	4.36	10.53	0.754	0.029	0.001	≤0.001	0.028
12th—rib fat (mm)	P1	Obs	4.0	2.03	7.5	1.2	-	-	-	-	-	-	-	-	-	-	-	-
		Norm + DMC	4.3	2.09	8.7	1.2	0.52	0.74	0.88	0.92	0.359	0.73	0.19	0.190	2.04	0.08	0.30	1.67
	P2	Obs	5.1	2.41	9.2	1.2	-	-	-	-	-	-	-	-	-	-	-	-
		Su, + 2^a^d	3.7	3.56	9.9	-2.0	0.54	0.63	3.25	0.64	≤0.05	0.52	0.13	0.003	7.23	2.19	2.75	2.28
L*	P1	Obs	33.4	5.86	38.2	19.0	-	-	-	-	-	-	-	-	-	-	-	-
		Au + Su	32.8	3.84	38.2	26.9	0.13	0.44	9.31	15.66	0.569	0.73	0.47	0.591	25.1	0.4	0.9	23.8
	P2	Obs	31.6	7.01	37.5	20.6	-	-	-	-	-	-	-	-	-	-	-	-
		Su	32.3	4.07	36.8	24.3	0.22	0.47	1.32	15.67	0.934	0.94	0.48	0.898	32.0	0.5	0.1	31.4
a*	P1	Obs	17.1	1.49	19.3	15.0	-	-	-	-	-	-	-	-	-	-	-	-
		SNV + Au	15.8	5.12	23.8	7.4	0.03	0.19	15.39	1.58	≤0.05	0.11	0.10	≤0.05	22.1	1.6	18.7	1.7
	P2	Obs	21.3	6.70	32.1	13.5	-	-	-	-	-	-	-	-	-	-	-	-
		Norm + Det	20.0	6.80	33.7	11.2	0.38	0.65	8.21	5.12	0.144	0.66	0.24	0.195	29.4	1.9	4.9	22.6

^1^pHu = carcass pHu 24hrs after slaughter, L* = luminosity, a* = red intensity;

^2^SA = spectra acquisition, P1 = spectra acquired on the center of the transversal cut of Longissimus dorsi muscle; and P2 = spectra acquired on the subcutaneous fat cap;

^3^Au. = Autoscaling, 1st d = first derivative, LB = baseline correction, Su. = smoothing, DMC = multiplicative scattering correction, Norm. = normalization, CM = center on the mean, SNV = standardized signal normalization, 2nd d = second derivative, Det. = detrend;

^4^standard deviation;

^5^coefficient of determination;

^6^coefficient of correlation and agreement or reproducibility index;

^7^standard error;

^8^H0: β0 = 0,

^9^H0: β1 = 1;

^10^mean square of prediction error;

^11^bias;

^12^magnitude of random fluctuation;

^13^random fluctuation of the model.

**Table 4:** Mean and descriptive statistics for the relationship between the observed and predicted values of carcass traits and meat quality of beef cattle (continued).

Parameters ^1^	SA^2^	Itens^3^	Average	SD^4^	Maximum	Minimum	R2^5^	CCC^6^	Regression: Intercept			Regression: Slope			MSEP^10^	QV^11^	MaF^12^	MoF^13^
									Estimated	SE^7^	P-value^8^	Estimated	SE	P-value^9^				
b*	P1	Obs	14.0	1.18	15.6	12.2	-	-	-	-	-	-	-	-	-	-	-	-
		Au + Det	14.8	3.28	19.5	9.5	-0.08	0.13	12.87	1.89	≤0.05	0.07	0.12	≤0.05	10.2	0.7	8.3	1.2
	P2	Obs	16.5	6.14	28.5	11.1	-	-	-	-	-	-	-	-	-	-	-	-
		DMC + Det	16.0	4.96	29.4	12.7	0.51	0.73	1.57	4.82	0.753	0.93	0.29	0.822	15.0	0.2	0.1	14.7
CL (%)	P1	Obs	1.4	0.31	1.9	0.7	-	-	-	-	-	-	-	-	-	-	-	-
		1^a^d + LB	1.4	0.10	1.5	1.2	0.01	0.16	0.23	1.17	0.848	0.82	0.83	0.828	0.082	0.001	≤0.001	0.081
	P2	Obs	1.4	0.24	1.9	1.1	-	-	-	-	-	-	-	-	-	-	-	-
		Norm + Det	1.5	0.12	1.7	1.3	-0.01	0.19	0.60	0.83	0.482	0.54	0.57	0.430	0.058	0.005	0.003	0.050
Total losses (%)	P1	Obs	20.4	3.43	27.8	14.3	-	-	-	-	-	-	-	-	-	-	-	-
		Det + CM	20.4	2.56	25.2	14.3	0.03	0.22	14.11	7.74	0.093	0.31	0.38	0.091	13.3	≤0.001	2.9	10.4
	P2	Obs	21.1	3.97	30.2	14.8	-	-	-	-	-	-	-	-	-	-	-	-
		2^a^d + LB	19.5	1.93	21.7	16.1	0.39	0.46	-5.50	8.70	0.539	1.37	0.45	0.424	11.4	2.8	0.4	8.2
Toughness (N)	P1	Obs	4.8	1.29	7.8	3.2	-	-	-	-	-	-	-	-	-	-	-	-
		Au + SNV	5.3	0.42	5.7	4.8	-0.06	0.07	2.58	4.68	0.591	0.42	0.88	0.522	1.82	0.24	0.05	1.53
	P2	Obs	4.3	2.73	7.8	1.3	-	-	-	-	-	-	-	-	-	-	-	-
		Su + DMC	1.9	2.08	5.7	4.1	0.62	0.70	-2.04	1.65	0.245	1.38	0.32	0.261	0.410	0.008	0.048	0.352

^1^b* = intensity of yellow, CL = cooling losses,

^2^SA = spectra acquisition, P1 = spectra acquired on the center of the transversal cut of Longissimus dorsi muscle; and P2 = spectra acquired on the subcutaneous fat cap;

^3^Au. = Autoscaling, Det. = detrend, 2nd d = second derivative, DMC = multiplicative spread correction, 1st d = first derivative, LB = baseline correction, Norm. = normalization, CM = center on the mean, Su. = smoothing, SNV = standardized signal normalization;

^4^standard deviation;

^5^coefficient of determination;

^6^coefficient of correlation and agreement or reproducibility index;

^7^standard error;

^8^H0: β0 = 0;

^9^H0: β1 = 1;

^10^mean square of prediction error;

^11^bias;

^12^magnitude of random fluctuation;

^13^random fluctuation of the model.

Moderate to high precision and accuracy (0.40 ≤ R2 ≤ 0.67 and 0.40 ≤ CCC ≤ 0.81) were observed when pHu, 12th-rib fat, and L* were estimated by models developed from the spectra acquired at P1, and when 12th-rib fat, L*, a*, b*, total losses, and toughness were predicted by models built from P2 spectra. Moreover, the prediction errors for pHu, 12th-rib fat, L*, CL, total losses, and toughness estimated from P1 spectra, as well as pHu, L*, a*, b*, CL, total losses, and toughness predicted from P2 spectra were mostly (more than 65% of MSEP) associated with random errors (MoF), rather than central tendency (SB), and due to linear regression or systematic bias (MaF).

However, poor accuracy and precision were observed when estimating a*, b*, CL, total losses, and toughness with P1 spectra models, and for predicting pHu and CL with P2 spectra models (R2 ≤ 0.27 and CCC ≤ 0.03). In addition, most of the error for 12th-rib fat from P2 spectra (more than 68.4% of the MSEP) was associated with central tendency (SB) and linear regression or systematic bias (MaF).

### 3.2. Composition of the section between the 9^th^ and 11^th^ ribs

For all 9th to 11th rib section constituents, the regression models obtained utilizing pre-treated spectra presented the lowest values of RMSECV, indicating the best model fit and precision (Table 5).

**Table 5:** Information on data sets used to develop and the respective performance parameters of PLS models to predict the chemical composition of the section between the 9^th^ and 11^th^ ribs of beef cattle.

Fraction	Spectra acquisition^1^	Parameters^2^	Pre-treatments^3^	Matrix size	nVL^4^	RMSECV^5^	RCV^6^
Meat plus Fat	P1	DM	CM + 2^nd^d	60x256	1	4.23	0.25
		EE	Su + Norm	59x256	3	7.90	0.22
		MM	Norm + 2^nd^d	60x256	1	0.57	-0.09
		CP	Det + 2^nd^d	60x256	1	8.87	-0.09
	P2	DM	DMC + Su	59x256	4	4.39	0.28
		EE	Au + 2^nd^d	60x256	2	6.47	0.62
		MM	2^nd^d + Su	58x256	2	0.57	-0.03
		CP	Au + 1^st^d	59x256	2	6.80	0.28
Bones	P1	DM	1^st^d + Norm	55x256	4	2.50	0.68
		EE	CM + LB	60x256	3	6.21	0.51
		MM	Norm + Det	60x256	1	3.37	-0.42
		CP	LB + SNV	60x256	10	0.34	0.73
	P2	DM	Au + Norm	58x256	2	3.22	0.50
		EE	2^nd^d + Norm	60x256	5	5.02	0.46
		MM	Norm + DMC	55x256	1	2.75	0.09
		CP	Au + LB	60x256	4	1.83	0.40
Meat plus Fat plus Bones	P1	EE	Su + CM	60x256	6	4.41	0.72
		MM	Norm + 1^st^d	59x256	1	2.74	-0.43
		CP	Au + Su	58x256	4	5.89	0.68
	P2	EE	SNV + 2^nd^d	59x256	6	4.71	0.54
		MM	Su + Norm	57x256	4	2.63	0.34
		CP	Norm + 1^st^d	58x256	6	5.56	0.62

^1^P1 = spectra acquired on the center of the transversal cut of Longissimus dorsi muscle; and P2 = spectra acquired on the subcutaneous fat cap,

^2^DM = dry matter, EE = ethereal extract, MM = mineral matter, CP = crude protein,

^3^size of the array used to generate the models,

^4^CM = center on the mean, 2^nd^d = second derivative, Su = smoothing, Au = autoscaling, 1^st^d = first derivative, Norm = normalization, Det = detrend, LB = baseline correction, SNV = standardized signal normalization, DMC = multiplicative scattering correction,

^5^number of latent variables,

^6^ root mean square error of the cross-validation,

^7^correlation coefficient of cross validation,

^8^ root mean square error of the calibration and ^7^correlation coefficient of calibration.

The models developed for estimating the MM and CP of the meat plus fat fraction from spectra acquired at both P1 and P2, as well as those models predicting the EE of the bones fraction from spectra acquired at both P1 and P2 presented moderate values of RMSECV in relation to the respective mean (22.56% ≤ RMSECV ≤ 31.2%). All other models of the chemical composition of all evaluated fractions obtained through the spectra acquired at both evaluated points of the section between the 9th and 11th ribs presented low values of RMSECV (RMSECV ≤ 17.5%). Normalize, smoothing, and second or first derivatives were used in 10, 6 and 11, respectively, of all 22 models developed for the chemical composition of all 9th to 11th rib section fractions evaluated from the spectra obtained at P1 and P2.

The predictive models for the DM, MM, EE, and CP constituents of the meat plus fat fraction showed low precision (–0.09 ≤ RVC ≤ 0.25) when developed from the spectra obtained at P1; whereas those developed from P2 spectra showed low precision (–0.03 ≤ RVC ≤ 0.28), with the exception of the model for EE, which showed moderate precision (RCV = 0.62) in the cross-validation. Models developed for predicting the contents of DM, MM, EE, and CP of the bone fraction presented moderate precision (RCV = -0.42 for MM and 0.51 ≤ RVC ≤ 0.73 for DM, EE and, CP) when developed from the spectra obtained at P1. Furthermore, the model predicting the MM of the bone fraction presented low precision (RVC = 0.09) when developed from P2 spectra; whereas those developed to estimate the DM, CP, and EE contents from spectra acquired at P2 exhibited moderate precision (0.40 ≤ RVC ≤ 0.50) in the cross-validation.

The models developed to estimate the EE and CP content of meat plus fat plus bone of the 9th to 11th rib section from the spectra acquired at both P1 and P2 showed moderate to high precision (0.62 ≤ RVC ≤ 0.72); whereas low precision (RVC = 0.34) was observed when the MM content was estimated by the model generated from the spectra acquired at P2, and moderate precision (RVC = -0.43) in the cross-validation when acquired at P1.

External evaluation analysis showed that predictive models developed from spectra acquired at P1 accurately estimated all chemical content (DM, EE, MM and, CP) of the 9^th^ to 11^th^ rib section constituents (meat plus fat, bones and meat plus fat plus bones) as the null hypothesis of a respective intercept and slope equal to 0 and 1 (P ≥ 0.066) was not rejected (Tables 6–8). Furthermore with the exception of the EE of the meat plus fat and bones fractions and the CP of the bones fraction, the models built from the spectra acquired at P2 accurately estimated the chemical constituents of the fractions evaluated as they did not reject (P ≥ 0.075) the null hypothesis of a respective intercept and slope equal to 0 and 1 (Tables 6–8).

**Table 6:** Mean and descriptive statistics for the relationship between the observed and predicted values of data used to develop models to predict the chemical composition of the fraction meat plus fat of the section between the 9th and 11th ribs of beef cattle.

Parameters^1^	SA^2^	Items^3^	Average (%)	SD^4^	Maximum	Minimum	R2^5^	CCC^6^	Regression: Intercept			Regression: Slope			MSEP^10^	QV^11^	MaF^12^	MoF^13^
									Estimated	SE^7^	P-value^8^	Estimated	SE^9^	P-value				
DM	P1	Obs	40.0	5.34	49.2	31.7	-	-	-	-	-	-	-	-	-	-	-	-
		CM + 2^nd^d	40.9	1.81	43.8	37.1	-0.07	-0.05	49.5	33.3	0.161	-0.2	0.8	0.154	31.740	0.664	1.813	26.434
	P2	Obs	40.8	4.99	49.8	33.6	-	-	-	-	-	-	-	-	-	-	-	-
		DMC + Su	40.8	2.77	45.9	35.5	0.21	0.44	2.9	17.5	0.872	0.9	0.4	0.875	17.094	0.004	0.033	17.055
EE	P1	Obs	63.2	8.32	78.9	50.7	-	-	-	-	-	-	-	-	-	-	-	-
		Su + Norm	64.0	4.95	71.7	55.2	-0.07	0.03	59.1	29.9	0.070	0.1	0.5	0.066	85.350	0.688	20.093	64.569
	P2	Obs	63.3	9.43	78.9	43.2	-	-	-	-	-	-	-	-	-	-	-	-
		Au + 2^nd^d	60.0	8.21	70.7	42.0	-0.01	0.24	45.5	18.6	0.029	0.3	0.3	0.039	119.448	10.876	31.096	77.475
MM	P1	Obs	2.2	0.54	3.0	1.4	-	-	-	-	-	-	-	-	-	-	-	-
		Norm + 2^nd^d	2.1	0.04	2.2	2.0	0.11	0.05	-9.6	7.2	0.203	5.7	3.5	0.197	0.259	0.017	0.030	0.212
	P2	Obs	2.1	0.57	3.0	1.3	-	-	-	-	-	-	-	-	-	-	-	-
		2^nd^d + Su	2.2	0.27	2.7	1.8	0.03	0.24	0.6	1.2	0.619	0.7	0.6	0.558	0.293	0.011	0.007	0.274
CP	P1	Obs	40.3	8.77	53.9	28.7	-	-	-	-	-	-	-	-	-	-	-	-
		Det + 2^nd^d	41.1	0.94	43.1	39.8	-0.01	-0.05	135.7	105.1	0.219	-2.3	2.6	0.215	77.480	1.971	1.400	66.766
	P2	Obs	39.7	12.74	64.1	23.7	-	-	-	-	-	-	-	-	-	-	-	-
		Au + 1^st^d	39.2	5.24	48.8	31.4	0.05	0.24	7.4	25.0	0.771	0.8	0.6	0.786	135.070	0.292	0.790	133.987

^1^DM = dry matter, EE = ethereal extract, MM = mineral matter and CP = crude protein;

^2^SA = spectra acquisition, P1 = spectra acquired on the center of the transversal cut of Longissimus dorsi muscle; and P2 = spectra acquired on the subcutaneous fat cap;

^3^CM = center on the mean, 2^nd^ d = second derivative, DMC = multiplicative spread correction, Su = smoothing, Norm = normalization, Au = Autoscaling, Det = detrend, 1^st^d = first derivative,

^4^standard deviation;

^5^coefficient of determination;

^6^coefficient of correlation and agreement or reproducibility index;

^7^standard error;

^8^H0: β0 = 0;

^9^H0: β1 = 1;

^10^mean square of prediction error;

^11^bias;

^12^magnitude of random fluctuation;

^13^random fluctuation of the model.

**Table 7:** Mean and descriptive statistics for the relationship between the observed and predicted values of data used to develop models to predict the chemical composition of the fraction bones of the section between the 9th and 11th ribs of beef cattle.

Parameters^1^	SA^2^	Items^3^	Average (%)	SD^4^	Maximum	Minimum	R2^5^	CCC^6^	Regression: Intercept			Regression: Slope			MSEP^10^	QV^11^	MaF^12^	MoF^13^
									Estimated	SE^7^	P-value^8^	Estimated	SE^9^	P-value				
DM	P1	Obs	61.3	3.98	69.9	56.0	-	-	-	-	-	-	-	-	-	-	-	-
		1^st^d + Norm	62.2	3.09	68.7	56.5	0.38	0.62	8.8	17.5	0.624	0.8	0.3	0.590	9.340	0.748	0.213	8.380
	P2	Obs	64.9	4.34	73.2	57.7	-											
		Au + Norm	62.8	2.27	65.9	59.0	-0.05	0.10	47.4	32.9	0.173	0.3	0.5	0.191	24.185	4.466	2.509	17.209
EE	P1	Obs	19.8	7.14	28.9	10.5	-	-	-	-	-	-	-	-	-	-	-	-
		CM + LB	21.6	7.82	39.1	9.1	0.44	0.67	5.6	4.1	0.186	0.7	0.2	0.070	35.891	3.424	6.696	25.489
	P2	Obs	24.1	7.76	34.1	11.4	-	-	-	-	-	-	-	-	-	-	-	-
		2^nd^d + Norm	18.6	7.75	29.8	3.1	0.09	0.32	16.7	5.1	≤0.05	0.4	0.3	≤0.05	97.647	30.052	20.283	47.312
MM	P1	Obs	51.4	4.05	57.3	42.6	-	-	-	-	-	-	-	-	-	-	-	-
		Norm + Det	52.7	0.05	52.7	52.6	-0.07	≤0.00	-269.5	1167.3	0.821	6.1	22.2	0.821	15.615	1.167	0.058	14.389
	P2	Obs	51.2	2.33	55.2	46.9	-	-	-	-	-	-	-	-	-	-	-	-
		Norm + DMC	53.6	1.43	55.6	51.4	0.17	0.24	9.0	22.1	0.691	0.8	0.4	0.615	9.720	5.770	0.060	3.860
CP	P1	Obs	31.5	2.70	36.2	26.9	-	-	-	-	-	-	-	-	-	-	-	-
		LB + SNV	29.4	2.27	33.2	26.6	0.25	0.41	12.4	8.1	0.146	0.6	0.3	0.213	8.740	3.740	0.580	4.419
	P2	Obs	29.1	3.22	34.4	21.7	-	-	-	-	-	-	-	-	-	-	-	-
		Au + LB	31.7	3.88	43.0	27.2	-0.07	0.06	28.8	9.7	≤0.05	0.1	0.3	≤0.05	28.577	6.795	7.847	13.935

^1^DM = dry matter, EE = ethereal extract, MM = mineral matter and CP = crude protein;

^2^SA = spectra acquisition, P1 = spectra acquired on the center of the transversal cut of Longissimus dorsi muscle; and P2 = spectra acquired on the subcutaneous fat cap;

^3^1^st^d = first derivative, Norm = normalization, Au = Autoscaling, CM = center on the mean, LB = baseline correction, 2^nd^ d = second derivative, Det = detrend, DMC = multiplicative spread correction, SNV = standardized signal normalization,

^4^standard deviation;

^5^coefficient of determination;

^6^coefficient of correlation and agreement or reproducibility index;

^7^standard error;

^8^H0: β0 = 0;

^9^H0: β1 = 1;

^10^mean square of prediction error;

^11^bias;

^12^magnitude of random fluctuation;

^13^random fluctuation of the model.

**Table 8:** Mean and descriptive statistics for the relationship between the observed and predicted values of data used to develop models to predict the chemical composition of the fraction meat plus fat plus bones of the section between the 9th and 11th ribs of beef cattle.

Parameters^1^	SA^2^	Items^3^	Average (%)	SD^4^	Maximum	Minimum	R2^5^	CCC^6^	Regression: Intercept			Regression: Slope			MSEP^10^	QV^11^	MaF^12^	MoF^13^
									Estimated	SE^7^	P-value^8^	Estimated	SE^9^	P-value				
EE	P1	Obs	42.0	10.39	62.8	29.0	-	-	-	-	-	-	-	-	-	-	-	-
		Su + CM	42.4	6.92	54.8	32.2	0.56	0.73	-3.7	10.4	0.727	1.1	0.3	0.756	43.188	0.219	0.327	42.642
	P2	Obs	42.0	10.58	64.2	29.8	-	-	-	-	-	-	-	-	-	-	-	-
		SNV + 2^nd^d	44.4	8.71	60.7	32.7	0.86	0.89	-8.4	5.4	0.145	1.1	0.1	0.276	19.980	5.497	1.306	13.476
MM	P1	Obs	15.6	2.95	21.0	10.8												
		Norm + 1^st^d	15.8	0.01	15.8	15.8	-0.08	≤0.01	75.0	1012.4	0.942	-3.8	64.0	0.941	8.510	0.236	0.001	8.247
	P2	Obs	15.1	2.58	19.5	10.8												
		Su + Norm	15.6	2.95	20.0	10.4	0.76	0.89	3.1	1.9	0.123	0.8	0.1	0.075	2.043	0.227	0.434	1.381
CP	P1	Obs	39.7	9.97	57.3	22.7	-	-	-	-	-	-	-	-	-	-	-	-
		Au + Su	40.9	5.84	46.6	29.6	0.42	0.59	-8.2	14.7	0.589	1.2	0.4	0.614	56.408	0.387	1.127	54.893
	P2	Obs	44.9	11.61	57.3	22.7	-	-	-	-	-	-	-	-	-	-	-	-
		Norm + 1^st^d	39.5	10.74	55.5	21.0	0.76	0.78	7.4	5.8	0.228	1.0	0.1	0.734	57.849	29.152	0.262	28.434

^1^DM = dry matter, EE = ethereal extract, MM = mineral matter and CP = crude protein;

^2^SA = spectra acquisition, P1 = spectra acquired on the center of the transversal cut of Longissimus dorsi muscle; and P2 = spectra acquired on the subcutaneous fat cap;

^3^Su = smoothing, CM = center on the mean, SNV = standardized signal normalization, 2^nd^ d = second derivative, Norm = normalization, 1^st^d = first derivative, Au = Autoscaling;

^4^standard deviation;

^5^coefficient of determination;

^6^coefficient of correlation and agreement or reproducibility index;

^7^standard error;

^8^H0: β0 = 0;

^9^H0: β1 = 1;

^10^mean square of prediction error;

^11^bias;

^12^magnitude of random fluctuation;

^13^random fluctuation of the model.

Low to moderate precision and accuracy (–0.07 ≤ R2 ≤ 0.44 and –0.05 ≤ CCC ≤ 0.62) were observed when DM, EE, MM, and CP were estimated by models developed from the spectra acquired at P1 and P2 of the meat plus fat and bones fractions. Moreover, the models build to predict the chemical composition (EE, MM, and CP) of the meat plus fat plus bones fraction showed high precision and accuracy (0.76 ≤ R2 ≤ 0.86 and 0.78 ≤ CCC ≤ 0.89) when the spectra were acquired at P2; whereas those developed from P1 spectra showed low to moderate precision and accuracy (-0.08 ≤ R2 ≤ 0.56 and 0.01 ≤ CCC ≤ 0.73).

The prediction errors for DM, EE, MM, and CP of the meat plus bones fraction estimated from P1 and P2 spectra; DM, EE, and MM of the bones fraction estimated from P1; EE, MM, and CP of the meat plus fat plus bones fraction estimated from P1; and EE and MM of the meat plus fat plus bones fraction estimated from P2 spectra were mostly (more than 64% of MSEP) associated with random errors (MoF), rather than central tendency (SB) and linear regression or systematic bias (MaF). However, most prediction errors for the EE, MM, and CP of the bones fraction estimated from P2; the CP of the bones fraction estimated from P1; and the CP of the meat plus fat plus bones fraction estimated from P2 spectra (more than 49.4% of the MSEP) were associated with central tendency (SB) and linear regression or systematic bias (MaF).

## 4. Discussion

### 4.1. Carcass and meat quality attributes

Various methods can be used to reduce model errors through mathematical operations, improving a model’s goodness of fit [27]. The use of spectral treatments that correct random and systematic errors [28] in the development of predictive models is an example of such methods. About 75% of the models developed to predict carcass and meat quality attributes from P1 spectra used autoscaling, alone or combined with another treatment. Autoscaling involves a combination of mean center and scaling by variance [29], detaching the spectral variation of the data [30]. The frequent use of the autoscaling in development of the carcass and meat quality attributes predictive models may have been due to the slight variation in sample compositions, making it necessary to make a better distinction among the spectra.

Values of a* and b* were not accurately or precisely estimated by the models developed from P1 spectra. Observed values for a* and b* in the dataset utilized for evaluating models developed from P1 varied little (average = 17.5, minimum = 12.9, and maximum = 32.2 for a*; average = 15.6, minimum = 11.1, and maximum = 31.0 for b*), increasing the significance of differences when there were small numerical variations between observed and predicted values and reducing model precision. The low variation in a* and b* may have been the resulted of removing outliers and separating the calibration and external evaluation datasets, as more outliers were identified and removed in from these datasets (two and one samples were removed as outliers for a* and b* in P1, respectively).

Moreover, models built from P2 spectra had problems adjusting for 12th-rib fat according to the intercept and inclination analysis, as well as the MSEP decomposition, more than 68.4% of the MSEP was associated with SB and Maf. This adjustment problem may have been due to the low association between the 12th-rib fat and the spectra collected in P2.

Bonin et al. [31] described low values of RMSECV (RMSECV ranged from 1.37 to 1.48) and moderate values of RCV (RCV ranged from 0.36 to 0.51) when predicting the toughness of the *Longissimus dorsi* muscle on the hanging carcasses of 401 Nellore steers. Despite the small sample size (n = 60), values of RMSECV and RCV (RMSECV ranged from 1.06 to 1.39 and RCV ranged from 0.10 to 0.51) observed in the current study were similar to those reported by Bonin et al. [31], regardless of the point of spectra acquisition (P1 or P2). Furthermore, the results of the external evaluation indicated that the models developed from P1 and P2 spectra accurately estimated the toughness of Nellore cattle meat (bulls and cull cows), with high precision and accuracy (R2 = 0.62 and CCC = 0. 70) being observed for the model built from P2 spectra.

### 4.2. Composition of the section between the 9^th^ and 11^th^ ribs

In general, most predictive models of the chemical composition of the meat plus fat fraction of the 9th to 11th rib section developed from spectra acquired at P2 presented better goodness of fit than those built from P1 spectra, as they presented a lower RMSECV and higher RVC. In addition, the model of CP content of the meat plus fat plus bones fraction built from the spectra acquired at P2 showed better goodness of fit in the cross-validation. However, the EE content of the meat plus fat plus bones fraction and all chemical constituents of the bones fraction were better predicted using P1 spectra. Nevertheless, all predictive models presented problems in adjustments for MM content, except the model built for the meat plus fat plus bones fraction from P2 spectra, regardless of the point of spectra acquisition (P1 or P2).

Dixit et al. [32] evaluated the contents of EE, CP, and MM in samples of fresh ground beef. The RMSECV and RCV for predicted contents of EE and CP described by these authors ranged from 3.50 to 6.54 and from 0.95 to 0.99, respectively, for EE content, whereas the variation of the same values for CP ranged from 1.29 to 2.19 for RMSECV and from 0.91 to 0.97 for RCV. The RMSECV values obtained for the EE content in the meat plus fat and meat plus fat plus bones fractions from both P1 and P2 spectra were similar to that observed in previous research (RMSECV ranged from 6.47 to 7.90 for meat plus fat and from 4.41 to 4.71 for meat plus fat plus bones). However, a lower value of RMSECV for the CP content was observed by Dixit et al. [32] when compared to those described for the CP content of meat plus fat (RMSECV ranged from 6.80 to 8.87) and meat plus fat plus bones (RMSECV ranged from 5.56 to 5.89) fractions in the current study, regardless of the spectra acquisition point (P1 or P2). In addition, the predictive model of the MM content in fresh ground beef proposed by Dixit et al. [32] showed superior predictive capacity (RMSECV ranged from 0.03 to 0.06 and RVC ranged from 0.95 to 0.99) when compared to the models proposed in the present study for predicting the MM content of the section between the 9th and 11th ribs, especially for the bone (RMSECV ranged from 2.75 to 3.37 and RVC ranged from -0.42 to 0.09) and meat plus fat (RMSECV of 0.57 and RVC ranged from -0.09 to -0.03) fractions. According to Kombolo-Ngah et al. [13], the inferior predictive capacity of models where the spectra were collected from intact samples can be attributed to the greater heterogeneity of those samples when compared to ground samples, such as those used by Dixit et al. [32]. Nonetheless, it is important to highlight that the small sample size was one of the limitations of the current study.

The NIR measures the absorption of radiation by organic molecular bonds. Thus, models for predicting the MM content may have inferior goodness of fit when related to models for predicting the content of organic materials, such as CP and EE. However, the correct prediction of the MM content, or even specific minerals, by NIR may be possible through the association of the minerals with the organic matrix of materials [29]. Hence, the higher proportion of bones, and consequently, of MM present in the bones and meat plus fat plus bone fractions may explain the inferior goodness of fit of such predictive models of MM content in relation to those predictive models of MM in the meat plus fat fraction, as well as to those models developed by Dixit et al. [32] to estimate the MM content in ground beef.

On the other hand, muscle fibers in unprocessed meat samples can produce greater internal reflection and light absorption (less reflectance) than processed and homogenized samples [33]. Thus, the destruction of the beef muscle fiber structure by grinding [34] reduces internal reflection in relation to intact fibers [35], which may result in models with better goodness of fit. Thus, the lack of sample processing before the acquisition of spectra (*in natura* and unprocessed samples) may also have contributed to the reduced goodness of fit of prediction of the MM content of the 9th to 11th rib section in the present study.

It is noteworthy that predictive models of the chemical composition of the bone and meat plus fat fraction (DM, EE, MM, and CP) were not precise (R2 ranged from –0.07 to 0.38 for DM; R2 ranged from –0.07 to 0.44 for EE; R2 ranged from –0.07 to 0.17 for MM; and R2 ranged from 0.01 to 0.25 for CP), regardless of the point of spectra acquisition (P1 or P2). In this context, the determination of the composition of the whole section between the 9th and 11th ribs would only be possible through the predictive models of the meat plus fat plus bone fraction.

Moreover, models built from P1 spectra had problems adjusting for CP in the bones fraction as did models built from P2 for EE, MM and CP in the bones fraction, and CP in the meat plus fat plus bones fraction according to the intercept and inclination analysis, as well as the MSEP decomposition (more than 49.44% of the MSEP were associated with SB and Maf). The adjustment problems for these variables may have affected the precision and accuracy of the models built from P2 for EE and, CP (P ≤ 0.01 and R2 ≤ 0.09).

The results of the external evaluation indicated that the models developed from P2 spectra accurately estimated the EE, MM and CP of Nellore cattle meat (bulls and cull cows), and that high precision and accuracy (0.76 ≤ R2 ≤ 0.86, and 0.78 ≤ CCC ≤ 0.89) were observed for the model built from P2 spectra.

However, to enhance the accuracy and robustness of the models predicting the chemical composition of the 9th to 11th rib section and carcass and meat quality attributes, further studies with larger sample sizes and increased variation in the origin of the samples are warranted.

Using NIR to predict the chemical composition and the carcass and meat quality attributes of *in natura* and unprocessed samples may be an important advance for the beef cattle production chain, especially for the meat industry, since it does not require sample collection and processing before performing the analysis, and there is no loss of meat that could be marketed.

The predictive models developed from spectra acquired at different points of the *in natura* and unprocessed section between the 9th and 11th ribs accurately estimated the carcass and meat quality attributes of beef cattle. The NIR spectra acquired at different points from *in natura* and unprocessed sections between the 9th and 11th ribs of Nellore cattle have the potential to be used as a tool to predict their chemical composition. Thus, this method could replace conventional analyses that are lengthy, laborious, and expensive. Nevertheless, it is important to highlight that the small sample size was one of the limitations of this present study. Further studies with a larger sample size are necessary to improve the precision and robustness of the predictive equations of the composition of the section between the 9th and 11th ribs, as well as to develop predictive equations for other animal categories.
